# Density and population size estimates of the endangered northern yellow-cheeked crested gibbon *Nomascus annamensis* in selectively logged Veun Sai-Siem Pang National Park in Cambodia using acoustic spatial capture-recapture methods

**DOI:** 10.1371/journal.pone.0292386

**Published:** 2023-11-27

**Authors:** Sarah J. McGrath, Jing Liu, Ben C. Stevenson, Alison M. Behie

**Affiliations:** 1 School of Archaeology and Anthropology, The Australian National University, Acton, ACT, Australia; 2 Department of Statistics, University of Auckland, Auckland, New Zealand; Salim Ali Centre for Ornithology and Natural History, INDIA

## Abstract

Many gibbon species are threatened with extinction, including the endangered northern yellow-cheeked crested gibbon, *Nomascus annamensis*. Assessing gibbon populations and understanding how human disturbances and environmental factors impact these populations is vital for effective conservation planning. In 2010, auditory surveys revealed that Veun Sai-Siem Pang National Park (VSSP) in Cambodia contains one of the largest known *N*. *annamensis* populations in the world, with an estimated 456 (95% CI 421–490) gibbon groups. Illegal selective logging is common in the park, but the impact of continued logging on the gibbon population has not been investigated. To determine any change in the *N*. *annamensis* population since 2010, between January and April 2019 we conducted auditory surveys at 13 sites that were at least 4 km apart. We surveyed each site for three days, each day recording the gibbon calls heard over 3.25 hours from three listening posts located 500 m apart. At the same sites, we assessed the logging intensity using transects and ecological plots. Gibbon densities can be influenced by various environmental factors such as canopy height and forest type. Therefore, in addition to investigating the relationship between the density of *N*. *annamensis* groups and logging, we included five additional environmental variables in our acoustic spatial capture-recapture models. Our best fit model with the lowest AIC value included canopy height, forest type, distance to villages, and logging. We estimate that there are 389 (95% CI 284–542) *N*. *annamensis* groups currently in VSSP. Selective logging is widespread in the park, primarily targeting four tree species. The estimated felling time of these logged trees, together with previous reports, indicate that the species most targeted in VSSP varies over time. To conserve the *N*. *annamensis* population in VSSP, it is crucial that action is taken to reduce illegal logging.

## 1 Introduction

In the tropics, insufficient knowledge of a species’ distribution and ecology frequently impedes conservation actions [1]. Between 2000 and 2012, 2.3 million km^2^ of forest was lost worldwide and in the tropics, forest loss increased by 2,101 km^2^ per year [2]. Declines in some primate populations have been reported following logging, however, the overall resilience of primate species to logging and other habitat disturbances varies [3–7]. The effect of logging on primate populations is influenced by numerous factors, such as the availability of food in the area, and the species’ degree of dietary flexibility [7] and arboreality [3]. Selective logging may increase visibility of arboreal primates, thus increasing predation risk. Due to a reduction in feeding trees and available travel routes in the canopy, arboreal primate species are typically more severely impacted by habitat disturbance [8].

Gibbons (Hylobatidae) are one of the most threatened primate families [9] as nearly all species are critically endangered or endangered [10]. These arboreal and territorial small apes [11] are found in the forests of Southeast Asia and in parts of South and East Asia [12, 13]. Most of the wild gibbon groups studied have consisted of an adult male and female [14], and up to four offspring [15], but groups with more than two adults have been observed in some species [14, 16–18]. Gibbons are hunted for food [19, 20] and traditional medicine, are captured for use in the pet trade [19–21], and are particularly vulnerable to forest degradation, fragmentation and loss [22]. Important factors that can affect the density and distribution of gibbons include forest type, elevation, distance from villages, and canopy height, cover and connectivity [1, 8, 23–28]. While some gibbon species have demonstrated the ability to tolerate logging through shifts in diet and behaviour [29], the impact of logging on gibbon density varies [3, 30, 31].

Accurately assessing and monitoring gibbon densities is crucial for their conservation [9] and when these surveys occur frequently, changes in populations due to anthropogenic disturbances can be detected [32]. All gibbon species produce loud calls known as songs [33], with most species producing male-female duets [34]. The duets, which last approximately 10–30 minutes [35], can be heard up to 2 km away [36] and typically occur early in the morning [33, 35]. Gibbon duets are species-specific [37], sex-specific [38] and may have several functions including strengthening of pair bonds [39], intergroup spacing [40] and territory defence [38, 41, 42]. Detecting gibbons visually in the field is difficult as they live high in the forest canopy and seldom descend to the forest floor [27, 29, 43]. Hylobatids are also extremely vigilant and act unpredictably to the presence of humans [27]. Therefore, gibbon density and population size are commonly estimated using auditory sampling methods instead of line transect methods [8, 23, 24, 27, 36, 43–46].

The crested gibbons (*Nomascus*) inhabit tropical evergreen and semi-evergreen forests [35] in Lao PDR (Laos), Vietnam, Cambodia and small parts of southern China [37, 47]. *Nomascus* gibbons are the most rare and one of the least studied [12] of the four gibbon genera [48]. Five of the seven crested gibbon species are critically endangered and two are endangered [10]. The endangered northern yellow-cheeked crested gibbon (*Nomascus annamensis*) is found in southern Laos, north-eastern Cambodia and central and southern Vietnam [43], east of the Mekong River, and was only described in 2010 [49]. These gibbons are largely frugivorous [50, 51], live in small groups with an average size of 2–5 individuals, and are thought to have territories of more than 0.3 km^2^ [46]. Hunting is a major threat to *N*. *annamensis* populations in Laos [52] and Vietnam [53], but the hunting pressure on the populations in Cambodia is lower [54, 55].

Assessments of *N*. *annamensis* populations are needed across the species’ range. In Laos, data on the densities and population sizes of the species is limited. A crude estimate of 400–6,720 groups in Xe Pian National Protected Area was obtained from surveys in 1992–1993 [56, 57], but this gibbon population has since declined [56]. The largest known population in Vietnam, an estimated 443 (95% CI 278–707) groups, resides in Song Thanh Nature Reserve [26]. Other areas have populations of 27–66 groups, yet in some areas of Vietnam where *N*. *annamensis* groups have been detected, the density and population size have not yet been estimated [26, 57].

Large *N*. *annamensis* populations are also found in Cambodia [58, 59]. However, Cambodia is experiencing high rates of forest loss [2, 60], threatening the country’s high level of biodiversity [61]. Between 2001 and 2021, Cambodia lost 26,000 km^2^ of tree cover [62] and 33.5% of the forest cleared between 2001 and 2015 was evergreen forest, the dominant forest type in the country [60]. Protected areas in Cambodia cover a large area [63, 64], yet many of these areas are not protected efficiently [64]. Consequently, primate populations are being affected by human activities, such as illegal logging, habitat degradation, and hunting [64]. Economic land concessions (ELCs) are one of the key drivers of deforestation in Cambodia [65]. ELCs occur both inside and outside of protected areas [60] and have resulted in substantial illegal logging outside of approved concession area boundaries [66].

In Cambodia, *N*. *annamensis* populations are located in Siem Pang Wildlife Sanctuary, Virachey National Park and Veun Sai-Siem Pang National Park (VSSP) [51]. These populations were previously thought to be additional populations of the southern yellow-cheeked crested gibbon *Nomascus gabriellae* [35]. However, following genetic and vocal analyses, the populations north of the Srepok River in Cambodia were reclassified as *N*. *annamensis* [35, 47, 49]. Virachey National Park (3,325 km^2^) has an estimated gibbon density of approximately 2.21 groups per km^2^ and population size of approximately 5,750 groups [59]. VSSP (14°01’N 106°44’E) is situated at the southern boundary of Virachey National Park. In 2012, using auditory survey data from 2010, it was reported that there were an estimated 456 (95% CI 421–490) *N*. *annamensis* groups in VSSP [58]. These data were then analysed using spatial capture-recapture methods by Kidney et al. [46], who estimated that on any given day, the density of calling gibbon groups per km^2^ was 0.32 (95% CI 0.19–0.49). Given the land area of VSSP is approximately 575 km^2^, we can extrapolate to an estimate of 184 (95% CI 109–282) gibbon groups calling on a given day during their study.

The resources within VSSP are important for the livelihoods of local people, providing food, income, and traditional medicine to those living in nearby villages [67, 68]. The hunting pressure on *N*. *annamensis* in VSSP is low, which may be due to a shortage of appropriate equipment, such as commercial guns, as there is high demand for the species for use as a pet [54]. Local people extract timber for building houses and income [68], and in VSSP, illegal selective logging has been occurring since the time of the previous *N*. *annamensis* survey in 2010 (N. Hon, pers. comm., October 31, 2022). However, no further gibbon population surveys have been conducted in the park to determine if illegal selective logging is impacting the size of this critical *N*. *annamensis* population.

In this study we aim to (1) estimate the density and population size of *N*. *annamensis* using acoustic spatial capture-recapture methods and (2) assess the intensity of logging at various locations and identify the most frequently logged trees in VSSP. Moreover, we aim to (3) ascertain the peak duetting time for *N*. *annamensis* in VSSP and (4) determine the relationship between gibbon density and six environmental variables: canopy height, forest type, elevation, and distance to rivers or streams, villages, and logging.

## 2 Methods

### 2.1 Study site

We conducted this study in VSSP, located in Ratanakiri Province and Stung Treng Province, between January 11 and April 27, 2019. The 574.69 km^2^ park, part of the Indo-Burma hotspot [61], was a Conservation Area until 2016 [63] and consists primarily of semi-evergreen and evergreen forest, with areas of bamboo forest, deciduous forest and grasslands. VSSP experiences a dry season during November–April and wet season during May–October [51] and the elevation ranges between 80 and 520 m asl. Between 1991 and 2020 in Ratanakiri Province, there was less rainfall and a lower mean temperature experienced in January (15.88 mm, 23.39°C) compared to April (63.91 mm, 28.21°C) [69]. There was a similar trend observed in the adjacent Stung Treng Province (January: 5.86 mm, 24.84°C; April: 69.26 mm, 29.55°C) [69].

Since the previous gibbon auditory survey, different tree species have been targeted for illegal selective logging in VSSP. In 2012, the most targeted luxury timber species were *Afzelia xylocarpa* and two rosewood species *Dalbergia cochinchinensis* and *Dalbergia oliveri*, which were unsustainably logged primarily for furniture [58]. Once *D*. *oliveri* became rare in the park, logging of *D*. *cochinchinensis* increased [58]. In 2013 and 2014, the most logged tree species in a surveyed area within VSSP was *Pterocarpus macrocarpus*, and *Hopea* spp., *Sindora cochinchinensis* and *D*. *oliveri*, among others, were logged at much lower frequencies [70]. In addition to *N*. *annamensis*, there are five other primate species found in VSSP: the long-tailed macaque (*Macaca fascicularis*), northern pig-tailed macaque (*Macaca leonina*), red-shanked douc langur (*Pygathrix nemaeus*), Annamese silvered langur (*Trachypithecus margarita*) and pygmy slow loris (*Nycticebus pygmaeus*) [58, 70, 71].

### 2.2 Auditory surveys

**Fig 1:**
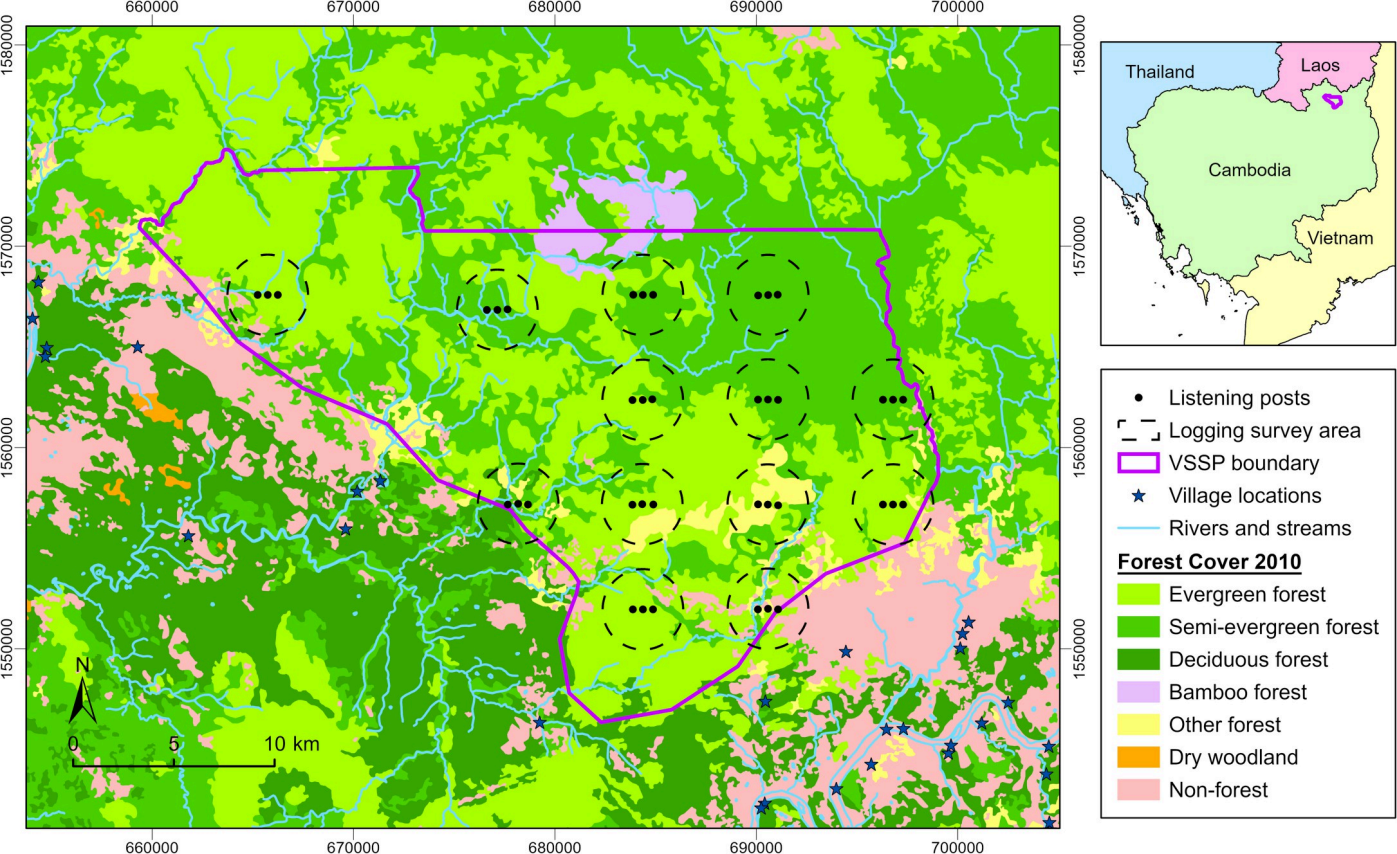
Map of the study area. Locations of the listening posts used for gibbon auditory surveys and the 2 km boundary for logging surveys in 13 sites in Veun Sai-Siem Pang National Park, Cambodia. Data for the following map components were provided by Conservation International: 1. forest cover (Forestry Administration (FA) and Ministry of Agriculture, Forestry and Fisheries (MAFF), 2010), 2. villages (updated by Conservation International, 2019; originally sourced from Japan International Cooperation Agency (JICA)), 3. rivers and streams (JICA, 2003) and 4. park boundary (Ministry of Environment (MoE), 2016).

Auditory surveys were completed by four trained researchers over the study period. Using a modified protocol from Kidney et al. [46], we used auditory sampling methods [36] at 13 sites in VSSP spaced at least 4 km apart. All but three listening posts were located at the same GPS locations as the 2010 survey [46] and these were positioned in a linear arrangement in each site, with 500 m between each of the three listening posts (Fig 1). As we were unable to access the same listening post locations in one site towards the northern VSSP boundary, we selected listening posts as close as possible, 1.3 km south-west from those used in the previous study. Since gibbon groups do not duet every day [72–74], gibbon auditory surveys are commonly conducted over three [8, 27, 75, 76] or four consecutive days in each site [23, 24, 44, 72]. We surveyed each site for three days to increase the number of gibbon groups heard, while maximising the overlap of survey dates with the previous study, which surveyed each site for one day during February and March in 2010 [46].

We surveyed each site for three consecutive days, except at one site, which we surveyed for three days over a four-day period (S1 Table) due to unforeseeable transportation issues. We surveyed three of our 39 listening posts for only two days (S1 Table). At the site where we were unable to access the same listening posts as in the 2010 survey, there was a sun bear at the location of one of the listening posts. We moved this post to the opposite end of the linear formation for day two and three of auditory surveying at that site (S1 Table). At another site, one researcher fell ill following the first day of surveying, therefore we conducted the survey at two of the three listening posts for two days instead of three.

We commenced the surveys 29–50 minutes before sunrise and recorded gibbon calls for 3.25 hours each day. Due to variation in sunrise times from 6:20am on January 18, 2019, to 5:33am on April 27, 2019, we surveyed sites 5:30–8:45am from January 18 to March 6, 2019, 5:15–8:30am from March 11 to April 9, 2019, and from 5:00–8:15am April 19 to 27, 2019. The researcher on the middle listening post recorded all chainsaw activity heard during the surveying period, scored on a noise intensity scale of one (loudest) to five (softest) and recorded the wind, temperature, and relative humidity at sunrise. The average temperature increased as the relative humidity decreased, with an average temperature of 17.68°C and relative humidity of 93.17% in January, whereas in April there was an average temperature of 25.08°C and relative humidity of 89.27% (S2 Table). No wind was detected from the middle listening post at any site. Light rain was recorded during the surveying period within 30 minutes of sunrise for four minutes at one site and for two minutes at another site. Additionally, rain at the latter fluctuated between medium and heavy 114–159 minutes after sunrise. As the medium to heavy rain occurred outside of the peak gibbon duetting time, these data were included in the analyses.

During the 3.25-hour auditory surveying period, we recorded the start and finish time and estimated the compass bearing of each gibbon call from each listening post. For a *N*. *gabriellae* population in a wildlife sanctuary in eastern Cambodia, the maximum hearing distance was estimated at 1.5 km [75]. The probability of detecting *N*. *annamensis* calling groups in VSSP at 2 km is approximately 0.3, lower than at 1.5 km [46]. Therefore, we estimated the distance of each *N*. *annamensis* calling group from each listening post up to 1.5 km using the following categories: 1) < 500 m, 2) 500 m, 3) 1000 m, and 4) 1500 m. We only used these estimated distances to differentiate groups as described in Section 2.4. We recorded if a male and female were both heard during the call and any repeat duets from a group. We also recorded any details that could help identify different groups, including the number of additional individuals heard during the female’s part of the duet. In *Nomascus* gibbons, the great call of the female may be terminated before it is completed [77], and the last part, known as the twitter phase, may not occur [35]. Therefore, we recorded whether this phase could be heard to aid in group identification. Furthermore, we recorded any gibbon calls that started or finished outside of the surveying period if part of the call was heard within the survey period.

### 2.3 Transects and ecological plots

We completed a maximum of three 500 m transects in six locations at each of the 13 sites. We selected the starting point for the central transect for each location at random within a 2 km radius of the middle listening post. To detect logging, transects were conducted by three researchers who were 40 m apart and travelled west using a GPS. The surveyed area was 62,400 m^2^ at each location, including 20 m surveyed from the end of each transect, therefore the maximum area surveyed per site was 374,400 m^2^. Once we found logging, if the stump circumference was ≥ 75 cm, we measured the height and diameter of the stump at the highest even point, recorded the parts remaining, and local research assistants estimated the felling time to the closest year and identified the species of the logged tree. We also recorded these measurements for other logged trees encountered while travelling throughout the site within the 2 km radius (Fig 1).

We selected this minimum stump circumference of 75 cm due to the number of logged trees seen while travelling, particularly along tracks, and the time available to measure logging. We were unable to record and measure every logged tree seen at each site outside of the surveyed area using transects, but this was supplementary data collected for descriptive statistics only. Importantly, we recorded and measured every logged tree that met this requirement along our transects and these data were used in our acoustic spatial capture-recapture (ASCR) models (Section 2.5). In cases where the diameter could not be accurately measured, these trees were included in the overall counts for each tree species (Table 1) but were excluded from calculations of the stump diameter mean and standard deviation (Table 4).

**Table 1:** The 18 most frequently logged trees species (N = 518) out of 635 logged trees recorded across 13 sites in Veun Sai-Siem Pang National Park, Cambodia, during January–April 2019.

Rank	Species	Local name	Frequency
1	*Pterocarpus macrocarpus*	Thnong	89
2	*Hopea* spp.	Koki	85
3	*Sindora cochinchinensis*	Ko koh	75
4	*Sterculia lychnophora*	Samrong	66
5	*Xylia dolabriformis*	So kram	36
6	*Dipterocarpus* spp.	Chheuteal	33
7	*Shorea cochinchinensis*	Popel	30
8	*Lagerstroemia calyculata*	Sra-lau	25
9	*Nephelium hypoleucum*	Se mornn	12
10	*Irvingia malayana*	Cham bak	12
11	*Peltophorum dasyrrhachis*	Trosek	11
12	*Lithocarpus elegans*	Khos / Krang	8
13	*Dipterocarpus intricatus*	Trach	8
14	*Dipterocarpus* sp. *(3*^*rd*^*)*	-	8
15	*Terrietia javanica*	Daunchem	**7**
16	*Fagraea fragrans*	Ta trav	5
17	*Ficus* sp.	-	4
18	*Syzygium lineatum*	Pring phnom	4
**Total**			**518**

Additionally, at each of the 13 sites, we completed a maximum of four 25 m × 25 m ecological plots. The location of each plot was chosen at random within a 2 km radius of the middle listening post at each site. Within each plot, we recorded the species’ name and diameter at breast height (DBH; 1.3 m) for bamboo and for all trees with a DBH ≥ 10 cm [78]. Where trees were multi-stemmed, we measured the DBH for each stem, then squared and summed each of these values, before taking the square root [79]. We recorded the aforementioned measurements for all logged trees located inside of the plots. Due to safety, dense vegetation, steep terrain, and time constraints, we could not complete all randomly selected transects and plots, despite choosing an order that would reduce travel time to maximise the number of each completed. We reselected plots if the location overlapped with another plot or if the plot was located inside farmland in VSSP. We reselected transect locations if the entire transect was not within the 2 km radius or if the surveyed area of two transects overlapped by ≥ 5%. When we could only partially complete a transect, we took a GPS location to determine the size of the area surveyed. We did not record tree fragments as logging unless there was at least a partial stump to ensure that it was not transported from elsewhere. Furthermore, we excluded logging found in farmland during transects, except in cleared land that had regenerated considerably.

### 2.4 Identification of gibbon groups

To determine the number of *N*. *annamensis* groups detected at each of the 13 sites, we first mapped the location of each group heard using the estimated bearing and distance. We identified the groups that called on a given day heard from one or more listening posts, and then determined which of these groups were also heard on other survey days. It is common for gibbon groups to be considered different groups when detections are located > 500 m apart [21, 25–27, 36, 44, 74]. In VSSP, *N*. *annamensis* groups typically duet around sunrise, when noise from other sources usually increases, such as from birds or cicadas. Neighbouring *N*. *annamensis* groups may even duet within 20 m of one another in overlapping areas of their home range or duet from a different area of their home range on different days (S. McGrath, pers. obs.). Furthermore, the inter- and intra-observer variation in recorded bearings becomes more problematic in detections further away, such as 1.5 km [9], and accurately estimating the direction of these detections can be particularly difficult in hilly terrain, as considerable echo of the gibbon duets can be produced.

In addition to the > 500 m criterion, we used several other factors to more accurately identify different groups. We also used the start and finish times of each duet, overlap of calling times, the proximity of the duet to each listening post in combination with which post heard each duet, and the recorded characteristics of the duet, outlined in Section 2.2. For example, in some instances, a group was heard by one listening post, on one survey day only. Even when the estimated location of the detection was more than 500 m from other detections, we did not identify this detection as a different group without first considering other factors. We would inspect the start and finish times of the duet and others on the same day to determine if this call: overlapped with other calls detected, occurred when many other calls were heard, matched the timing of a duet detected from another listening post, or was the only duet heard from all posts at that time. We would also compare the number of individuals and any noted duet characteristics of those heard. Furthermore, we used the estimated location of the detection to determine if another listening post was likely to hear that same duet.

We excluded repeat duets heard by gibbon groups on the same day from our analyses. Additionally, as the males may not be part of a mated pair [74], we excluded male solo calls from our analyses [27, 44–46, 80]. However, during our study, we found that for duets heard far away from a listening post, the adult male’s part of the duet was easier to hear than the female’s part. When it was evident from the factors outlined above that this detection was a duet rather than a male solo call (e.g., another listening post closer to that call detected a duet from the same location that started and stopped at the same time), we included these calls in our analyses.

### 2.5 Acoustic SCR

We estimated gibbon group density, abundance, and distribution using an acoustic spatial capture-recapture (ASCR) model. ASCR methods have been used in recent years to estimate the density of other primate species [24, 81]. In our study, in addition to estimating the density of the primate species at various sites within a national park, we also estimate the population size of the species in the entire park.

A particular advantage of ASCR over many competing approaches, such as distance sampling, is that we do not need to determine the locations of calling groups [82]. Instead, the true locations of calling groups are treated as latent variables and are integrated out of the likelihood function (see [82, 83]). Our approach was similar to that proposed by Stevenson et al. [83], who estimated call density of frogs using their ASCR model, which was converted to an estimate of population density by dividing by call rate. Here, we estimate daily calling group density using an ASCR model similar to Kidney et al. [46], which we convert to an estimate of the density of all groups by dividing by daily calling probability.

Our ASCR model requires daily detection records (or “capture histories”) for each detected group. For the *k*th gibbon group detected on the *j*th day of surveying at the *i*th set of listening posts, we observed a capture history **ω**_*ijk*_ = (ω_*ijk*1_,ω_*ijk*2_,⋯), where ω_*ijkl*_ = 1 if the *l*th listening post detected the group at some point during that ɷday, and ω_*ijkl*_ = 0 otherwise. For example, a capture history of (1,1,0) indicates that the group was detected by the first and second listening posts sometime that day, but not by the third. As we directly modelled daily group capture histories, our model estimated the density of calling groups on a given day (calling groups per day per km^2^). This component of our method did not require groups to be recognised across different days.

#### 2.5.1 The state process

The state process of our SCR model describes the abundance and distribution of daily gibbon group locations. We used the standard approach for SCR models and assumed that the locations of calling gibbon groups were a realisation of an inhomogeneous Poisson process [84]. The daily calling group density at the coordinates **s** = (*s*_*x*_,*s*_*y*_) is given by *D*(**s**), which is modelled using a linear combination of spatial environmental variables as follows:

$$log\{D(s)\}=\beta_{0}+\beta_{1}x_{1}(s)+\beta_{2}x_{2}(s)+\cdots ,$$ (1)

where *x*_1_(**s**) is the first spatial environmental variable measured at location **s**, *x*_2_(**s**) is the second, and so on.

The expected number of daily calling groups (TCG) within VSSP can be calculated from the state process:

$$TCG=\int_{V}\;D(s)\;ds,$$ (2)

where *V* is the region within the VSSP boundaries.

Numerous studies have illustrated that the density of gibbons can be influenced by various environmental factors [8, 23–26]. Therefore, it was essential that we include additional variables within our model, not only to increase our understanding of what influences *N*. *annamensis* density in VSSP, but also to account for possible confounding variables when determining the relationship between logging intensity and gibbon density.

We considered the effect of six environmental variables on daily calling group densities of *N*. *annamensis* in VSSP:

1. Distance to logged trees: we used the GPS coordinates (N = 168) of logged trees found along transects in this study, allowing us to calculate the distance from any location **s** to the nearest logged tree
2. Canopy height: we used 2019 raster data available online from [85]
3. Distance to villages: GPS coordinates of Cambodian villages allowed us to calculate the distance from any location **s** to the nearest village (data: updated by Conservation International (CI), 2019; originally sourced from Japan International Cooperation Agency (JICA))
4. Elevation: we used polyline data, elevation in VSSP ranged from 80 to 520 m asl (data: updated by Ministry of Environment (MoE), 2013; originally sourced from JICA)
5. Distance to the nearest stream or river: we used polyline data (data: JICA, 2003)
6. Forest type: we used 2010 data and three forest categories: (1) evergreen, (2) semi-evergreen, (3) non-forest and deciduous, bamboo and other forests. As this variable is categorical, we used one of the categories as a baseline, and included dummy variables for the other categories in the linear combination presented in Eq (1) (data: Forestry Administration (FA) and Ministry of Agriculture, Forestry and Fisheries (MAFF), 2010)

The data used for variables 3–6 were provided by CI and we used ArcGIS Pro 2.4.3 [86] to convert data for variables 4–6 to point data using a grid of 30 m × 30 m cells. Additionally, variable 2 was converted from raster data to point data.

We fitted 64 models covering all possible linear combinations of environmental variables. The model selection was conducted based on AIC. We initially considered interaction effects involving forest type, and separately nonlinear main effects of the environmental variables, but did not find any improvement in AIC, and so we did not consider these effects further. To carry out the calculation in Eq (2), we needed to observe any environmental covariates included in the model across the entirety of VSSP. Because we only recorded logged trees within a 2 km radius of the middle listening post at each site, and not throughout the entire VSSP, we could not estimate TCG using models that included distance to logged trees.

#### 2.5.2 The detection process

The detection process of our SCR model describes how gibbon groups are detected, given their daily locations. ASCR methods use a detection function, *g*(*d*), to model the probability of detection as a function of distance between a sound source and a detector: the probability of the *l*th detector detecting the *k*th group on the *j*th day of surveying at the *i*th set of listening posts, given the location of the group, **s**_*ijk*_, is therefore given by *P*(ω_*ijkl*_ = 1|**s**_*ijk*_) = *g*{*d*(**s**_*ijk*_,**t**_*ijl*_)}, where **s**_*ijk*_ is the location of the group, **t**_*ijl*_ is the location of the listening post, and *d*(**s**,**t**) is the distance between the locations **s** and **t**. Here we used the hazard halfnormal detection function, and so the probability that a calling gibbon group at location **s** is detected by a listening post at location **t** is given by:

$$g\{d(s,t)\}=1\;-\;exp\;\left\{-\lambda_{0}\;exp\left(\frac{-d{\left(s,t\right)}^{2}}{2\sigma^{2}}\right)\right\}$$ (3)

We used the hazard halfnormal detection function because it allows a ‘shoulder’ at probability 1, respecting that gibbon groups call sufficiently loud for detection to be virtually certain over some appreciable distance. The halfnormal detection function, the most common alternative, is guaranteed to drop away from 1 at any distance greater than 0, which is not a realistic scenario for our survey.

For each detection, we also observed the estimated bearing from the listening post to the gibbon call. We used the method of Borchers et al. [82] to include these data in our ASCR model. Borchers et al. [82] showed that incorporating estimated bearings in ASCR can dramatically improve precision of population density estimates. Their method accommodates measurement error in bearing estimates, thus it is not a requirement that the estimated bearings are exact, although we do assume that they are unbiased (e.g., errors in one direction are just as common as errors in the other). The method of Borchers et al. [82] estimates the magnitude of measurement errors via a parameter κ. A large κ indicates that bearing estimates are very precise and are likely to point towards the true locations of the detected gibbon groups, whereas κ = 0 indicates that bearing estimates are completely uninformative about gibbon group locations.

#### 2.5.3 Model fitting

Our models estimated the following parameters: the β parameters in Eq (1); the detection function parameters, λ_0_ and σ, in Eq (3); and the accuracy of bearing estimates, κ. The β parameter estimates allow us to investigate how daily calling density varies across the entire VSSP. To fit our model, we used the package ascr v2.2.3 [87] in R version 4.1.0 [88]. The only data we required for our model were the capture histories, the estimated bearings, the listening post locations, and the spatial environmental covariates. To deal with both uncertainty in calling group locations and the fact that we can only observe spatial covariates at discrete locations, a ‘habitat mask’ was needed [84]. A habitat mask is a fine grid of equally spaced locations throughout the survey region at which we require measurements of our environmental variables. We created a fine mask of 960,000 grid points across VSSP.

#### 2.5.4 Estimating daily calling probabilities and group density

Our ASCR model estimated *D*(**s**), the density of gibbon groups calling on any given day at any location **s** within VSSP. To estimate the density of all groups, we calculated group density at location **s** by dividing by the probability of a group calling on any given day, *p*, to give *D*_*g*_(**s**) = *D*(**s**)/*p*. We also calculated the total number of gibbon groups (TGG) in a similar way: *TGG* = *TCG*/*p*. This is an equivalent procedure to that described by Stevenson et al. [83] to convert from call density of frogs to population density. We used parametric bootstrapping to calculate the 95% confidence interval (95% CI) for the TCG and TGG.

To estimate daily calling probability, *p*, we used the 37 groups with a detection distance of ≤ 500 m from at least one listening post on at least one survey day (S3 Table). We determined that these groups were likely to remain close enough to our listening posts so that their daily call detection probabilities were very close to 1 across all days (i.e., if one of these groups call on any of our days of surveying, we are almost guaranteed to detect it), and that these groups could reliably be identified between different days. Therefore, for each of these groups, we could reliably construct a temporal detection history over the different days of our survey. For the *k*th such group, we observe **α**_*k*_ = (α_*k*1_,α_*k*2_,⋯), where α_*kj*_ = 1 if the group called on the *j*th day, and α_*kj*_ = 0 otherwise. For each group, we then calculated $y_{k}=\sum_{j=1}^{n_{k}}\alpha_{jk}$, the number of days on which the group called during the survey, where *n*_*k*_ is the number of days of surveying at the listening posts that detected the group.

If we assume that whether or not a group calls on a particular day is independent of whether or not it called on any other day, and that calling probability is constant over all days and groups, then for any given group, *y*_*k*_ ~ *Binomial*(*n*_*k*_,*p*). However, groups that do not call at all during the *n*_*k*_ days of surveying remain undetected and will not appear in our records, and so it is impossible to observe *y*_*k*_ = 0. We therefore assumed that *y*_*k*_ ~ *Zero*-*TruncatedBinomial*(*n*_*k*_,*p*), and estimated *p* by maximum likelihood. The value of *p*(*m*), the estimated proportion of groups expected to call during *m* days, is commonly used for estimating gibbon densities (e.g., [21, 23, 24]). It is calculated using the equation *p*(*m*) = 1 –[1 –*p*(1)]^*m*^, where *p*(1) is the calling probability on a given day and *m* is the number of survey days [36]. Similar to our methods, the assumption for this correction factor *p*(*m*) is that calling on successive days is independent [74].

### 2.6 Ethics statement

This research was approved by the Australian National University Animal Experimentation Ethics Committee (Wildlife Animal Ethics Protocol Number: A2015/14 and A2018/26) and did not involve capturing and handling non-human primates or any other wildlife. This study was conducted in the protected Veun Sai-Siem Pang National Park, Cambodia with permission from the General Department of Administration for Nature Conservation and Protection, Ministry of Environment, Cambodia (Letter number 196).

## 3 Results

### 3.1 Logged trees

Across the 13 sites, 635 logged trees were measured, 596 were identified to the local name or scientific name and 39 were unknown species to the research team (Table 1). The maximum area surveyed for logging along transects within a site was 374,400 m^2^ (Table 2). Of the 635 logged trees measured, 27.09% (172/635) were found in areas surveyed using transects, 32.56% (56/172) of which were logged ≤ 1 year ago (Table 3). *Pterocarpus macrocarpus* (22.09% 38/172), *Hopea* spp. (15.12%, 26/172), *Sindora cochinchinensis* (13.37%, 23/172) and *Sterculia lychnophora* (13.37%, 23/172) were the most encountered logged trees along transects. Similarly, the most encountered logged trees outside of the areas surveyed using transects were *Hopea* spp. (12.74%, 59/463), *P*. *macrocarpus* (11.02%, 51/463), *Sindora cochinchinensis* (11.23%, 52/463) and *S*. *lychnophora* (9.29%, 43/463).

**Table 2:** The total area surveyed for logged trees from transects and the number of logged trees (N = 172) found within the surveyed area in each site in Veun Sai-Siem Pang National Park, Cambodia, between January and April 2019.

Site	Area surveyed (m^2^)	Logged trees
1	312,000	37
2	374,400	31
3	306,000	13
4	312,000	14
5	88,000	3
6	258,120	23
7	312,000	19
8	157,080	1
9	62,400	0
10	279,480	29
11	0	-
12	124,800	2
13	312,000	0
**Total**	2,898,280	172

**Table 3:** The estimated felling time (years) of logged trees encountered within each site (N = 624 of 635) and those only found in the transect survey areas (N = 172) within the 13 sites in Veun Sai-Siem Pang National Park, Cambodia.

Estimated felling time	Transect logged trees	All logged trees
< 1y	44 (25.58%)	86 (13.78%)
1y	12 (6.98%)	56 (8.97%)
2y	22 (12.79%)	64 (10.26%)
3y	37 (21.51%)	155 (24.84%)
4y	12 (6.98%)	35 (5.61%)
5y	27 (15.70%)	171 (27.40%)
> 5y	18 (10.47%)	57 (9.13%)
**Total**	**172**	**624**

**Fig 2:**
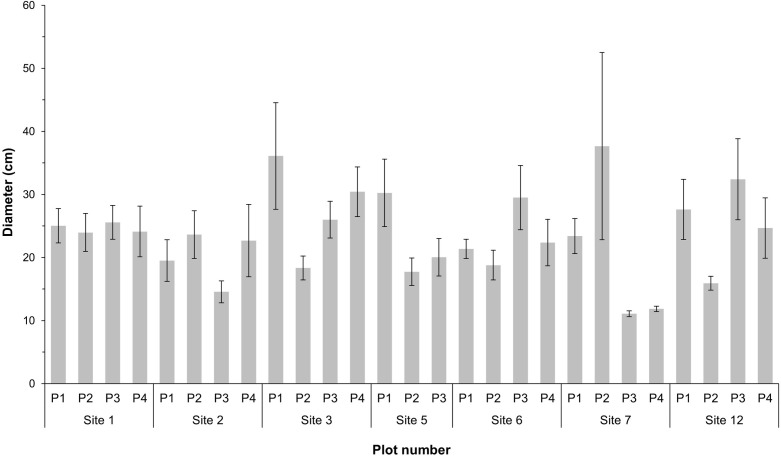
Tree DBH in ecological plots. The mean (± SE) diameter at breast height (DBH) of all trees with a DBH of ≥ 10 cm within 25 m × 25 m ecological plots (N = 27) in seven sites, located in Veun Sai-Siem Pang National Park, Cambodia.

Over 75% of *Hopea* spp., *Sindora cochinchinensis* and *S*. *lychnophora* logged trees were felled in the last three years, whereas 82.76% of *P*. *macrocarpus* were felled ≥ 4 years ago (Table 4). *Hopea* spp. logged trees had the largest mean (SD) diameter of 124.91 (62.42, 187.41) cm and *S*. *lychnophora* had the smallest, 42.23 (27.63, 56.82) cm (Tables 4 and S4). However, we excluded logged trees that could not be measured accurately, including numerous *P*. *macrocarpus* stumps which only remained in pieces and when only part of the *Hopea* spp. stump remained. We completed 27 ecological plots across seven of the 13 sites and at different sites, three logged trees were detected in one plot and one logged tree in another. The mean (SD) number of trees and bamboo ≥ 10 cm DBH in each site was 26 (19.84, 32.16) in site 1, 17 (6.83, 27.17) in site 2, 20.75 (17.88, 23.62) in site 3, 18 (14.54, 21.46) in site 5, 20 (15.45, 24.55) in site 6, 27.25 (17.34, 37.16) in site 7 and 21.50 (13.65, 29.35) in site 12. Bamboo with a DBH of ≥ 10 cm was detected within seven plots across three sites and the DBH of trees (includes bamboo) in plots varied within sites and between sites (Fig 2 and S5 Table).

**Table 4:** The estimated felling time, and stump height and diameter of the four most logged tree species encountered in 13 sites in Veun Sai-Siem Pang National Park, Cambodia between January and April 2019.

Species	*Pterocarpus macrocarpus*	*Hopea* spp.	*Sindora cochinchinensis*	*Sterculia lychnophora*
**Estimated felling time**	(N = 87)	(N = 84)	(N = 73)	(N = 66)
< 1–1 year	3.45%	26.19%	15.07%	81.82%
2–3 years	13.79%	66.67%	61.64%	7.58%
4–5 years	63.22%	5.95%	15.07%	10.61%
5+ years	19.54%	1.19%	8.22%	0%
**Stump height (cm)**	(N = 86)	(N = 84)	(N = 74)	(N = 66)
Mean ± SD	65.26 ± 25.16	126.79 ± 55.27	61.38 ± 25.97	95.00 ± 30.27
**Stump diameter (cm)**	(N = 56)	(N = 64)	(N = 73)	(N = 66)
Mean ± SD	80.32 ± 44.36	124.91 ± 62.50	88.27 ± 25.94	42.23 ± 14.60

### 3.2 Chainsaw activity

Chainsaws were heard from the middle listening post during the gibbon auditory survey period in six of the 13 sites (Table 5). The loudest chainsaws (level 1–2 on the 5-level noise intensity scale) were heard at two sites. At site 7, a level 2 chainsaw could be heard for five minutes on day three 28–39 minutes after sunrise. In site 4, a level 1 chainsaw was heard for 51 minutes on day two 77–157 minutes after sunrise, and a level 1–2 chainsaw was active for 26 minutes on day three 56–158 minutes after sunrise.

**Table 5:** The number of locations, duration (minutes), and intensity (1, loudest to 5, softest) of chainsaws heard in six of the 13 sites in Veun Sai-Siem Pang National Park, Cambodia, during the gibbon auditory surveys January–April 2019.

**Site**	**Day**	**Locations**	**Duration**	**Intensity**
**1**	3	1	6	5
**2**	1	4	30	4–5
	2	3	17	5
	3	5	53	5
**3**	2	1	14	4–5
	3	2	8	5
**4**	2	1	51	1
	3	1	26	1–2
**7**	2	2	26	5
	3	5	60	2–5
**10**	2	1	4	4–5

### 3.3 Peak duetting time of detected groups

**Fig 3:**
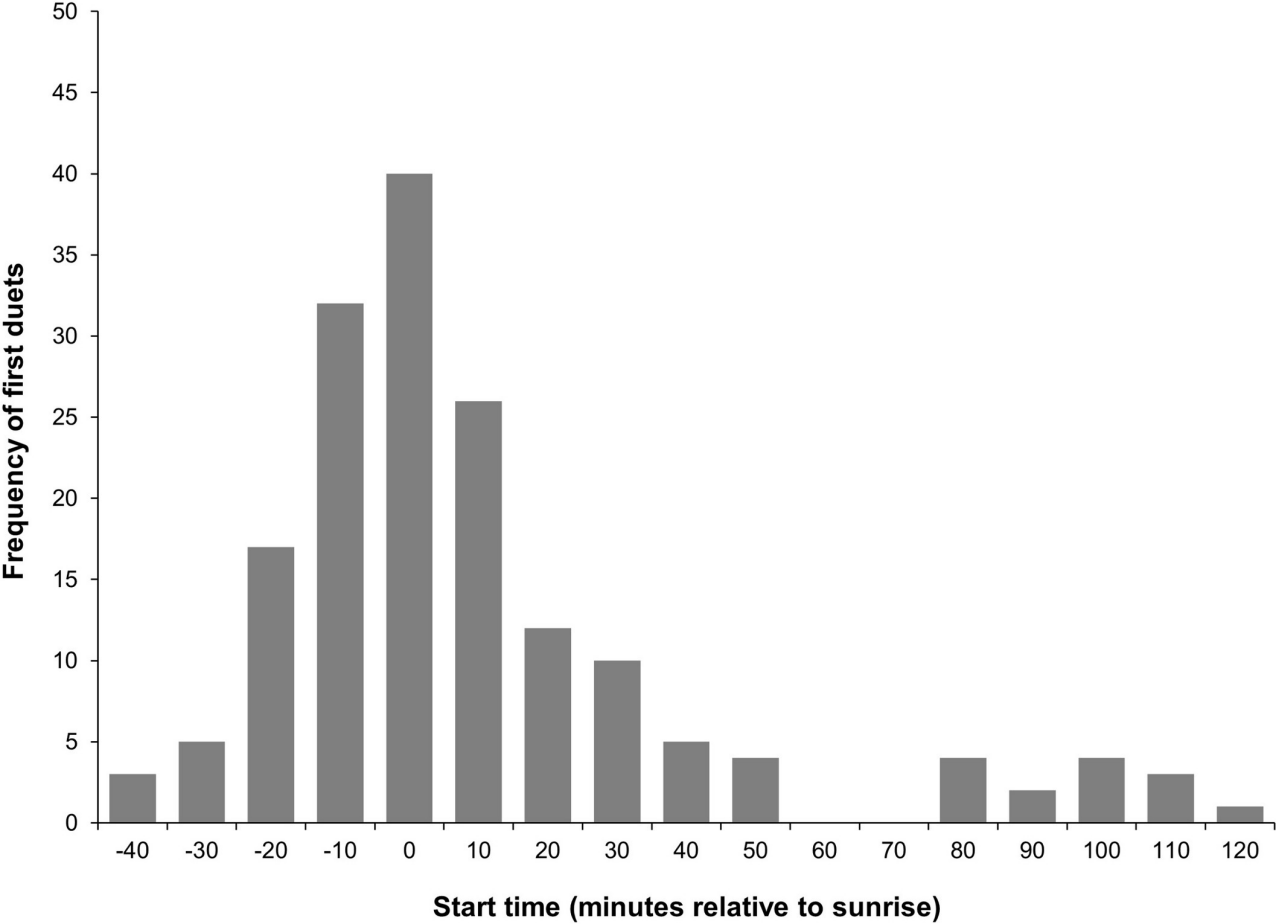
The start time of *N*. *annamensis* duets relative to sunrise. The frequency of the first duets (N = 168) from gibbon groups heard during each 10-minute block relative to sunrise in the auditory survey period in Veun Sai-Siem Pang National Park, Cambodia. The frequency of first duets (*x*) at start time ‘0’ are those that began -10 < *x* ≤ 0 minutes relative to sunrise.

Ninety-two *N*. *annamensis* groups were detected across the 13 sites in VSSP, 68 were heard by more than one listening post, and 55 groups were heard on at least two of the three survey days by one or more listening posts. The 10 minutes prior to sunrise was the most common 10-minute interval (23.81%, 40/168) for gibbon groups to duet (Fig 3 and S6 Table) and 81.55% (137/168) of first duets were heard within a one-hour period commencing from 30 minutes before sunrise. This excludes all repeat duets, except the second duet of one group on one day because the group’s first duet began 80 minutes before sunrise and lasted 10 minutes.

### 3.4 Acoustic SCR

Using our model selection procedure, we identified a four-variable model with the highest AIC support. This model included the variables forest type, distance to villages, canopy height, and distance to logging. Our estimates indicate that distance to villages, canopy height, and distance to logging are all positively associated with gibbon group density (Table 6). There was a significantly greater density of calling gibbon groups per day in Forest 1 evergreen forest compared to Forest 2 semi-evergreen forest (density coefficient β = 1.45, 95% CI = 0.56–2.33, *p* < 0.01). Our estimate corresponds to Forest 1 evergreen forest having estimated calling group densities that are 4.3 (95% CI 1.8–10.3) times as high as Forest 2 semi-evergreen forest, holding all other environmental variables constant. However, there was no significant difference in the density of calling gibbon groups per day in Forest 1 evergreen forest (β = 0.66, 95% CI = -0.33–1.65, *p* = 0.19) or Forest 2 semi-evergreen forest (β = -0.79, 95% CI = -2.06–0.48, *p* = 0.23) compared to Forest 3, non-forest and deciduous, bamboo and other forests.

**Table 6:** Output of the ASCR models illustrating the relationship between the density of calling *N*. *annamensis* groups and environmental variables: forest type (F), canopy height (CH), distance to villages (V) and logging (L), elevation (E) and distance to rivers or streams (R) in Veun Sai-Siem Pang National Park, Cambodia.

Model	AIC difference	Density coefficient (β)								p-value						TCG (95% CI)	TGG (95% CI)
		*Intercept*	CH	F2	F3	V	L	E	R	CH	F^	V	L	E	R		
**Full model**	2.68	-5.251	0.538	-1.338	-0.490	0.538	0.144	-0.104	*-*0.053	0.108	-	< 0.001*	0.211	0.528	0.754	-	-
**Best fit model (4 variables)**	0	-5.149	0.393	-1.445	-0.659	0.480	0.157	-	-	0.136	0.009*	< 0.001*	0.100	-	-	-	-
**3-variable model**	0.33	-5.266	0.402	-0.987	-0.632	0.443	-	-	-	0.131	0.022*	< 0.001*	-	-	-	251.24 (185.69, 330.40)	389.16 (284.19, 542.31)
**2-variable model**	2.24	-5.017	-	-1.282	-0.831	0.535	-	-	-	-	< 0.001*	< 0.001*	-	-	-	253.45 (184.60, 341.82)	392.59 (281.12, 555.78)
**1-variable model**	9.66	-5.545	0.737	-	-	-	-	-	-	< 0.001*	-	-	-	-	-	297.96 (214.63, 379.14)	461.53 (329.59, 631.27)
**No variables**	27.58	0.006	-	-	-	-	-	-	-	-	-	-	-	-	-	317.45 (228.99, 409.34)	491.72 (344.20, 675.84)

Forest categories are F1) evergreen (baseline), F2) semi-evergreen, F3) non-forest and deciduous, other and bamboo forests. AIC difference represents the difference in AIC values compared to the best fit model with the lowest AIC value of 1740.26. TCG and TGG represent the total number of gibbon groups calling on a given day and total number of gibbon groups estimated in VSSP respectively.

^^^ Forest type is a categorical variable. Therefore, we conducted a likelihood-ratio test to determine the significance of forest type (F).

The estimated daily calling probability of *N*. *annamensis* in VSSP was 0.646 (95% CI 0.545–0.756). As our best fit model included logging, a variable that is not available throughout the entire region (Section 2.5.1), we used our best three-variable model to estimate the TCG and TGG. This model included all other variables from the best fit model and only had slightly less AIC support (Table 6). Using this best three-variable model, the estimated average daily calling density was 0.437 calling gibbon groups per km^2^, the estimated TCG was 251 (95% CI 186–330; SE 36.67; CV 14.60%) groups and the estimated TGG was 389 (95% CI 284–542; SE 66.16; CV 17.00%) groups in VSSP.

**Fig 4:**
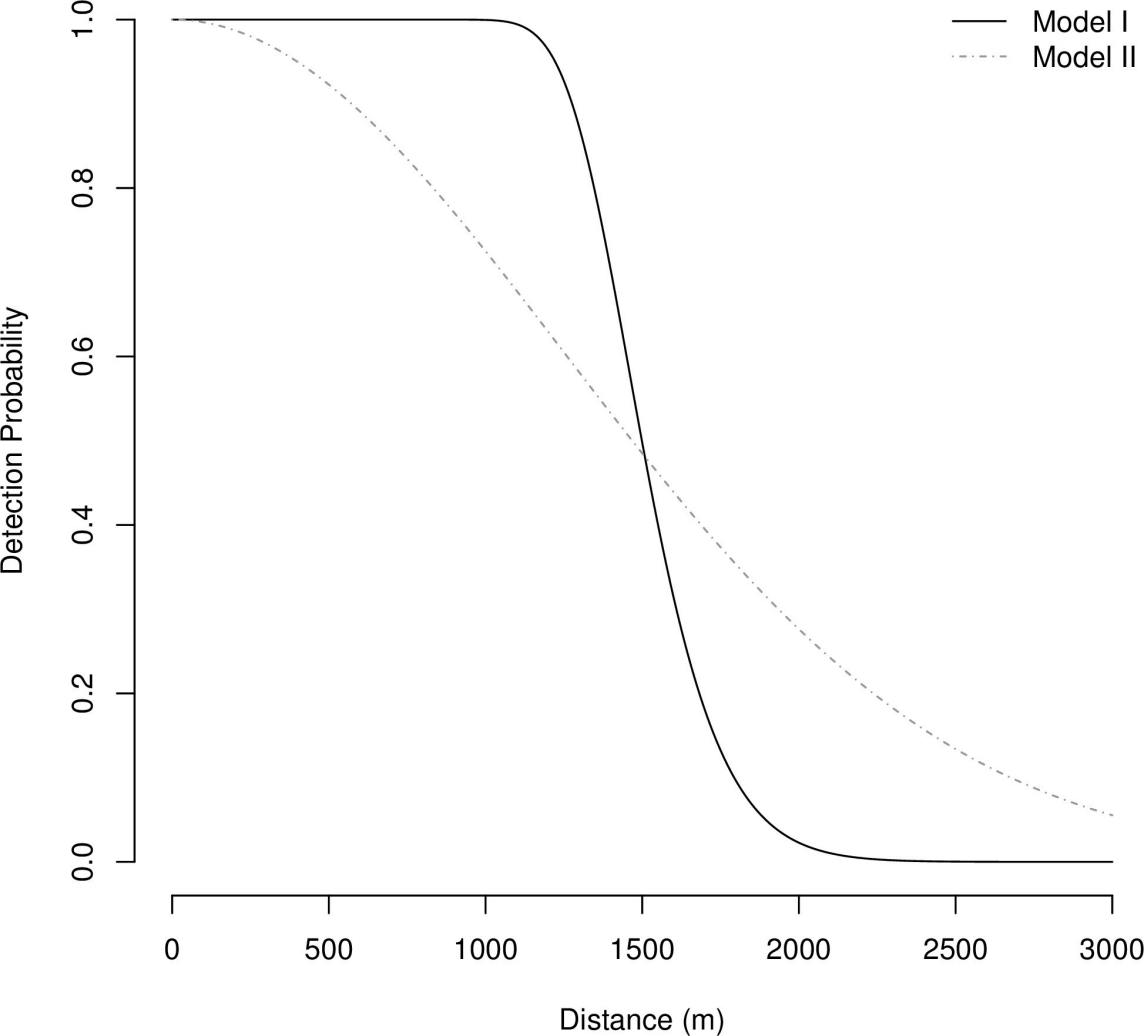
Detection function. The estimated hazard halfnormal detection function for our best fit model (Model I; λ = 54.55, σ = 507.29) and the estimated halfnormal detection function from Kidney et al. [46] (Model II).

From the detection function fitted by our best fit model (Fig 4), we estimate that a calling gibbon group within 1000 m of a listening post is detected with probability 1, and that detection is unlikely beyond 2000 m.

## 4 Discussion

### 4.1 Logging in VSSP

Our study demonstrates that illegal selective logging is frequently occurring within VSSP. Chainsaws were heard from numerous locations during the auditory surveying period in six sites. Recorded bearings and noise levels indicate that almost all chainsaws heard were located within the park boundary. Furthermore, a total of 635 logged trees were recorded across 12 of the 13 sites but many more logged trees were seen throughout the survey area and between sites, particularly near existing tracks. Logged *Pterocarpus macrocarpus*, *Hopea* spp., *Sindora cochinchinensis* and *Sterculia lychnophora* trees were encountered the most, and combined, accounted for half of the 635 logged trees recorded. Three of these species are large trees targeted for timber, whereas *S*. *lychnophora* is logged, using a chainsaw or an axe, for its fruit. According to local people involved in the logging trade, timber from VSSP is transported to Ban Lung, the Ratanakiri Province capital, then to Vietnam and China [68]. Shifts to other tree species have been reported when targeted species for logging become rare [58, 68]. González Monge [70] found that *P*. *macrocarpus* was felled the most in the surveyed area in VSSP during the study between 2013 and 2014. *Hopea* spp., *Sindora cochinchinensis* and *Dalbergia oliveri* were also logged during the study, but comparatively much less [70]. In our study, over 80% of *P*. *macrocarpus* logged trees were felled four or more years ago, whereas for the other three tree species, most of those detected were felled within the preceding three years. Our study demonstrates that there has been another shift in the trees being targeted for logging in VSSP, this time from *P*. *macrocarpus* to *Hopea* spp., *Sindora cochinchinensis* and *S*. *lychnophora*.

### 4.2 Peak duetting time

To our knowledge, our study is the first to record the peak duetting time of *N*. *annamensis* groups to a short time frame of 10 minutes. Approximately 80% of first duet calls from the 92 groups detected were heard during a one-hour period beginning 30 minutes before sunrise. Less than 5% of duets were heard between 30 and 50 minutes before sunrise and the last first duet call was heard approximately 110 minutes after sunrise. Auditory surveys occurred following a full moon on one occasion. The earliest duet of the entire survey period was heard on the following morning, 80 minutes before sunrise when the area was much brighter due to moonlight. Later that morning during the surveying period, the same group duetted again. This indicates that moonlight can influence the timing of *N*. *annamensis* duets.

The peak duetting time for *N*. *annamensis* is relatively similar to other *Nomascus* gibbons. In the Keo Seima Wildlife Sanctuary in Mondulkiri Province, Cambodia, 90% of *N*. *gabriellae* duets were heard within a one-hour period that commenced 10 minutes before sunrise [75]. However, in Nakai-Nam Theun National Park in Laos, a greater proportion of first duets commenced before sunrise than in our study, with most *Nomascus* sp. duets starting during the 20 minutes before sunrise [89]. Our findings suggest that a surveying time of three hours commencing 50 minutes before sunrise would likely capture a high proportion of *N*. *annamensis* first duets in conditions similar to our study site. Gibbon duetting behaviour can be influenced by other factors, including temperature [89], rain [41, 74, 75], wind [74, 75, 89] and hunting pressure [90]. Therefore, site-specific variables need to be considered when establishing the time frame for auditory surveys.

### 4.3 Relationship between environmental variables and gibbon density

We estimated an average density of 0.552 daily calling gibbon groups per km^2^ and population size estimate of 492 (95% CI 344–676) groups for VSSP from our model without any environmental variables. There is substantial overlap in the confidence intervals of this population size estimate and the estimate of 456 (95% CI 421–490) *N*. *annamensis* groups from the 2010 survey [58]. Therefore, we cannot conclusively state that the *N*. *annamensis* population size in VSSP has changed since that time. Additionally, when canopy height, forest type and distance to villages were incorporated into our best three-variable model, the estimated average density was 0.437 daily calling gibbon groups per km^2^ and the estimated TCG and TGG were 251 (95% CI 186–330) and 389 (95% CI 284–542) groups respectively.

The reason for the discrepancy between these two models, is that the model without environmental variables relies on the regions near the listening posts being representative of the entire VSSP when estimating TCG and TGG. However, once we incorporate environmental variables measured across the entire park, we can account for any differences in habitat suitability between regions near the listening posts and those that were not monitored. Estimates of TCG and TGG from our inhomogeneous density model were lower than those from our constant-density model, suggesting that the environmental variables in regions of the park that were not within listening distance of our listening posts had, on average, lower habitat suitability than those that were. A further advantage of using the environmental variables is that the additional information is reflected in more precise estimates of TCG and TGG in terms of CV. Previous studies that analysed data from auditory surveys in VSSP [46, 58] used constant-density models, however our study highlights the potential benefits of using environmental variables to estimate spatially varying gibbon group density instead. Additionally, in our study, the detection function illustrates that the probability of detecting *N*. *annamensis* calling groups in VSSP is much lower at 2 km than at 1.5 km. This is consistent with other studies, as although gibbon duets can typically be heard within 2 km [36], the maximum hearing distance for *Nomascus* gibbons has been estimated at 1.5 km (e.g., *N*. *gabriellae* [75]).

Our best fit model shows that *N*. *annamensis* density is related to forest type, canopy height, and distance to villages and logging in VSSP. We found that the estimated density of calling groups was higher with increasing canopy height and distance to logging and villages. The density of gibbons in the genus *Hylobates* have been found to increase with habitat quality [8, 25], vary in different forest types [28] and be positively correlated with tree height, canopy cover and large trees [23, 91]. Hankinson et al. [24] demonstrated that while the density of white-handed gibbons (*Hylobates lar*) was positively correlated with trees that were tall, large and had a large crown area, the density of siamangs (*Symphalangus syndactylus*) was not significantly correlated with those variables. However, both *S*. *syndactylus* and *H*. *lar* densities were positively correlated with the degree of canopy connectivity [24]. Furthermore, in the Central Annamite mountain range in Vietnam, habitat quality was shown to have a substantial impact on the occurrence probability of *N*. *annamensis* [92].

In Song Thanh Nature Reserve in Vietnam, the degree of habitat suitability for *N*. *annamensis* gibbons increased with distance from villages, and in rich and medium evergreen broad‐leaved forests [26]. The closely related [49] *N*. *gabriellae* inhabits areas dominated by evergreen, mixed deciduous, deciduous, semi-evergreen and bamboo forest [75]. A study conducted in a wildlife sanctuary in the Mondulkiri Province in Cambodia, demonstrated that the occupancy of *N*. *gabriellae* was greater in evergreen forest than in semi-evergreen forest [1]. Conversely, in another wildlife sanctuary in Mondulkiri Province, the density of *N*. *gabriellae* did not significantly differ between evergreen, semi-evergreen and deciduous forests, although their ability to persist in other forest types is thought to be influenced by the presence of evergreen patches therein [75]. Moreover, *N*. *gabriellae* groups living in bamboo-dominated forests may occupy much larger home ranges of up to 1 km^2^, than those living in evergreen forests (approximately 0.3 km^2^) [75].

In VSSP, there was a significantly higher density of *N*. *annamensis* calling groups in evergreen forest compared to semi-evergreen forest. However, there were no significant differences between both evergreen and semi-evergreen forest compared to the third forest type consisting of non-forest and deciduous, bamboo and other forests. The forest cover data used in our analyses represents locations where each forest type is dominant. For example, in our study, in a site that was categorised as part semi-evergreen and part evergreen forest, bamboo was recorded in three of the four plots. Additionally, there was variation in the number and DBH of trees in plots in the same site and between sites. In VSSP, there may be essential patches of evergreen forest present in areas dominated by forest types in the third forest category, except non-forest, that enable *N*. *annamensis* to inhabit those areas.

While *N*. *annamensis* inhabits areas located at a large range of elevations [57], the probability of occupancy decreases substantially in areas where elevation is higher than approximately 700 m asl [93]. The exclusion of elevation from our best fit model was not unexpected as elevation in VSSP ranges from 80 to 520 m asl. In the park, some of the sites that we surveyed were easier to access than others due to the presence of tracks suitable for tractors or motorbikes. Distance from park boundaries and roads have been shown to affect pileated gibbon (*Hylobates pileatus*) densities, which may be due to greater human disturbance and accessibility for hunting near those areas [8]. Proximity to tracks may be another important environmental factor contributing to differences in *N*. *annamensis* densities in the park. However, determining the location of these tracks within the park is difficult, as most of them are not visible in satellite images. Furthermore, over time, forest regeneration may impact the density and distribution of gibbon groups in VSSP.

In our study, the loudest chainsaws (level 1–2) were only heard for two minutes during peak duetting time, the one-hour period commencing 30 minutes before sunrise. Chainsaws low on the noise intensity scale were likely from areas outside of the site surveyed at the time. A study in VSSP found that the calling probability of *N*. *annamensis* was not impacted chainsaw activity [94]. It is possible that chainsaw noise may affect the calling probability of gibbons when it occurs within their home range. At one site, a chainsaw was active approximately 100 m away from a listening post. This activity was entirely outside of the peak duetting time, but a group less than 500 m away from that listening post began to duet three minutes after the chainsaw had temporarily stopped. This group continued to call for 13 minutes, even when the chainsaw activity resumed. Furthermore, at the same site, two more groups were heard on day three than on day two, despite chainsaw activity at the same location on day two after the peak duetting time. Therefore, these surveys were not repeated, and the data were included in our analyses.

### 4.4 Conservation implications

Our study demonstrates that the *N*. *annamensis* population in VSSP is still one of the largest known populations in the world. *Nomascus annamensis* also inhabits the adjacent Virachey National Park, with approximately 5,750 groups estimated in the 3,325 km^2^ park [59]. These populations are likely a global stronghold for the species; therefore, it is crucial that action is taken immediately to decrease logging to protect these gibbon populations. During selective logging, generally only a small percentage of trees are removed, but the damage to the area can be extensive [95]. The effect of logging on gibbon populations is varied, as studies have shown that gibbon densities may decrease, increase, or remain stable in the years after logging [3, 30, 31]. Changes in the diet of gibbons may also occur. For example, *H*. *lar* shifted to a more folivorous diet in logged forest compared to primary forest, due to a decrease in fruit availability [29]. In VSSP, selective logging is decreasing the availability of several known feeding trees. Out of the top 10 logged tree species, *N*. *annamensis* has been observed feeding on certain plant parts of *Shorea cochinchinensis* (S. McGrath, pers. obs.), *S*. *lychnophora*, *Irvingia malayana* as well as one of the three *Dipterocarpus* spp. known locally as Chheuteal [51].

Gibbons, including *N*. *annamensis* [96], select taller trees [97–100] with a greater DBH for sleeping [99, 100]. *Nomascus annamensis* also duets in taller trees [96] and it is primarily large, tall trees that are targeted by loggers in VSSP. Furthermore, the gaps in the canopy caused by selective logging may disrupt the travel routes of the gibbon groups. To access and remove these trees from VSSP, tracks are created that are wide enough for two-wheel tractors with long, flat trailers, known in Cambodia as koyun. Roads created in tropical forests for logging increases accessibility and the hunting pressure on wildlife [101–103]. The time taken for gibbons to recover from population declines is higher than other mammals [53] as the maturation period of gibbons is lengthy [8]. Even though many hunters in the villages surrounding VSSP lack suitable equipment for hunting gibbons [54], the tracks created by loggers will give hunters greater access to the gibbons, thereby increasing hunting pressure should suitable equipment become available in the area.

### 4.5 Conclusion

Using ASCR methods, we estimate that there are 389 (95% CI 284–542) *N*. *annamensis* groups in VSSP. Greater *N*. *annamensis* calling densities were estimated with increasing distance to villages and logging, and as canopy height increased. We also found evidence to suggest calling group densities are higher in evergreen forest than in semi-evergreen forest. Illegal selective logging is a widespread issue in the VSSP. As targeted trees are depleted within the park, loggers are shifting to other tree species. This may be due to changes in availability of the targeted tree species or changes in the demand for each species. We recommend that immediate and ongoing action be taken to reduce illegal logging, including more frequent patrols in the park by a greater number of rangers. Moreover, to help conserve this endangered species, we recommend further monitoring of the *N*. *annamensis* population in the future to detect any changes in the size of the population.

## Supporting information

S1 Table The UTM (zone 48N) coordinates of each listening post and the number of days each listening post was used for during *N*. *annamensis* surveys in 13 sites in Veun Sai-Siem Pang National Park, Cambodia, between January and April 2019. (PDF)

S2 Table The dates of the *N*. *annamensis* surveys, and relative humidity (%) and temperature (°C) recorded at the middle listening post at each of the 13 sites in Veun Sai-Siem Pang National Park, Cambodia. (PDF)

S3 Table The proportion of days that each *N*. *annamensis* group (N = 37) duetted during the auditory survey in Veun Sai-Siem Pang National Park from January to April 2019 to estimate the calling probability. These groups were selected from the 92 groups detected during the survey as they had a detection distance of ≤ 500 m from at least one listening post on at least one survey day.
(PDF)

S4 Table The stump height (cm) and diameter (cm) of the four most logged tree species encountered within 13 sites in Veun Sai-Siem Pang National Park, Cambodia, between January and April 2019. These data were used to calculate the mean (cm) and standard deviation (cm) values presented in Table 4.
(PDF)

S5 Table The diameter at breast height (DBH; cm) of all trees (including bamboo) with DBH of ≥ 10 cm within 25 m × 25 m ecological plots (N = 27) in seven sites, located in Veun Sai-Siem Pang National Park, Cambodia. These data were used to calculate the mean (cm) and standard error (cm) values presented in Fig 2.
(PDF)

S6 Table The start time of the first duet call heard from each *N*. *annamensis* group relative to sunrise. These data were used to determine the frequency of the first duets (N = 168) from gibbon groups heard during 10-minute blocks relative to sunrise in the auditory survey period in Veun Sai-Siem Pang National Park, Cambodia, presented in Fig 3. The frequency of first duets (*x*) in category ‘0’ are those that began -10 < *x* ≤ 0 minutes relative to sunrise.
(PDF)
