# Supplementary material for: Density and population size estimates of the endangered northern yellow-cheeked crested gibbon Nomascus annamensis in selectively logged Veun Sai-Siem Pang National Park in Cambodia using acoustic spatial capture-recapture methods
Source: PLoS One. 2023 Nov 27;18(11):e0292386. doi: 10.1371/journal.pone.0292386 (PMC10681233; doi:10.1371/journal.pone.0292386)
Supplement: S1 Table — (PDF) [file pone.0292386.s001.pdf]

S1 Table. The UTM (zone 48N) coordinates of each listening post and the number of days each listening post was used for during *N. annamensis* surveys in 13 sites in Veun Sai-Siem Pang National Park, Cambodia, between January and April 2019.

Site	Detector	Post #	X - UTM	Y - UTM	Usage	Day #
1	1A	1	683869	1551956	101	1, 2
	1B	2	684369	1551956	10101	1, 2, 3
	1C	3	684869	1551956	101	1, 3
2	2A	1	690078	1551956	10101	1, 2, 3
	2B	2	690580	1551986	10101	1, 2, 3
	2C	3	691078	1551956	10101	1, 2, 3
3	3A	1	677660	1557165	10101	1, 2, 3
	3B	2	678176	1557202	10101	1, 2, 3
	3C	3	678660	1557165	10101	1, 2, 3
4	4A	1	683848	1557157	10101	1, 2, 3
	4B	2	684336	1557183	10101	1, 2, 3
	4C	3	684885	1557167	10101	1, 2, 3
5	5A	1	690085	1557174	10101	1, 2, 3
	5B	2	690570	1557163	10101	1, 2, 3
	5C	3	691082	1557128	10101	1, 2, 3
6	6A	1	696287	1557165	10101	1, 2, 3
	6B	2	696787	1557165	10101	1, 2, 3
	6C	3	697287	1557165	10101	1, 2, 3
7	7A	1	683851	1562357	10101	1, 2, 3
	7B	2	684360	1562363	10101	1, 2, 3
	7C	3	684837	1562394	10101	1, 2, 3
8	8A	1	690084	1562380	10101	1, 2, 3
	8B	2	690578	1562383	10101	1, 2, 3
	8C	3	691078	1562369	10101	1, 2, 3
9	9A	1	696287	1562374	10101	1, 2, 3
	9B	2	696787	1562374	10101	1, 2, 3
	9C	3	697287	1562374	10101	1, 2, 3
10	10A	1	665242	1567583	10101	1, 2, 3
	10B	2	665742	1567583	10101	1, 2, 3
	10C	3	666242	1567583	10101	1, 2, 3
11	11A	1	676622	1566835	101	2, 3
	11B	2	677143	1566838	10101	1, 2, 3
	11C	3	677646	1566863	10101	1, 2, 3
12	12A	1	683890	1567588	10101	1, 2, 3
	12B	2	684367	1567582	10101	1, 2, 3
	12C	3	684875	1567586	10101	1, 2, 3
13	13A	1	690090	1567557	10101	1, 2, 3
	13B	2	690586	1567576	10101	1, 2, 3
	13C	3	691061	1567592	10101	1, 2, 3
