# Supplementary material for: Density and population size estimates of the endangered northern yellow-cheeked crested gibbon Nomascus annamensis in selectively logged Veun Sai-Siem Pang National Park in Cambodia using acoustic spatial capture-recapture methods
Source: PLoS One. 2023 Nov 27;18(11):e0292386. doi: 10.1371/journal.pone.0292386 (PMC10681233; doi:10.1371/journal.pone.0292386)
Supplement: S2 Table — (PDF) [file pone.0292386.s002.pdf]

S2 Table. The dates of the *N. annamensis* surveys, and relative humidity (%) and temperature (°C) recorded at the middle listening post at each of the 13 sites in Veun Sai-Siem Pang National Park, Cambodia.

Date	Site	Survey day	Humidity (%)	Temp (°C)
18-Jan-19	2	1	95.0	19.2
20-Jan-19	2	2	94.5	18.7
21-Jan-19	2	3	92.5	20.5
24-Jan-19	7	1	93.4	15.5
25-Jan-19	7	2	93.6	15.0
26-Jan-19	7	3	90.0	17.2
03-Feb-19	8	1	92.4	19.2
04-Feb-19	8	2	94.7	21.0
05-Feb-19	8	3	94.3	20.1
16-Feb-19	3	1	93.1	17.0
17-Feb-19	3	2	92.9	18.5
18-Feb-19	3	3	90.0	22.0
23-Feb-19	1	1	87.7	20.7
24-Feb-19	1	2	88.9	22.7
25-Feb-19	1	3	85.3	23.3
04-Mar-19	4	1	86.1	23.5
05-Mar-19	4	2	90.4	23.5
06-Mar-19	4	3	88.4	22.1
11-Mar-19	13	1	94.4	21.3
12-Mar-19	13	2	94.0	22.0
13-Mar-19	13	3	94.6	22.9
16-Mar-19	12	1	94.0	21.8
17-Mar-19	12	2	95.1	20.7
18-Mar-19	12	3	93.3	20.6
28-Mar-19	9	1	85.2	22.5
29-Mar-19	9	2	87.4	24.0
30-Mar-19	9	3	90.9	24.9
02-Apr-19	6	1	89.5	22.8
03-Apr-19	6	2	90.8	23.9
04-Apr-19	6	3	90.8	23.7
07-Apr-19	5	1	82.0	24.4
08-Apr-19	5	2	86.2	25.3
09-Apr-19	5	3	87.5	26.1
19-Apr-19	10	1	89.3	27.4
20-Apr-19	10	2	92.2	25.9
21-Apr-19	10	3	89.4	28.2
25-Apr-19	11	1	93.8	23.5
26-Apr-19	11	2	88.1	24.7
27-Apr-19	11	3	91.6	25.1
**Monthly averages (mean)**		Jan	93.2	17.7
		Feb	91.0	20.5
		Mar	91.2	22.5
		Apr	89.3	25.1
