# Supplementary material for: Density and population size estimates of the endangered northern yellow-cheeked crested gibbon Nomascus annamensis in selectively logged Veun Sai-Siem Pang National Park in Cambodia using acoustic spatial capture-recapture methods
Source: PLoS One. 2023 Nov 27;18(11):e0292386. doi: 10.1371/journal.pone.0292386 (PMC10681233; doi:10.1371/journal.pone.0292386)
Supplement: S3 Table — These groups were selected from the 92 groups detected during the survey as they had a detection distance of ≤ 500 m from at least one listening post on at least one survey day. (PDF) [file pone.0292386.s003.pdf]

S3 Table. The proportion of days that each *N. annamensis* group (N = 37) duetted during the auditory survey in Veun Sai-Siem Pang National Park from January to April 2019 to estimate the calling probability. These groups were selected from the 92 groups detected during the survey as they had a detection distance of ≤ 500 m from at least one listening post on at least one survey

Group	Day 1	Day 2	Day 3	Total	Proportion
1	1	1	0	2	0.667
2	1	1	0	2	0.667
3	0	0	1	1	0.333
4	1	1	0	2	0.667
5	1	1	1	3	1.000
6	1	1	1	3	1.000
7	1	1	0	2	0.667
8	1	0	0	1	0.333
9	0	1	1	2	0.667
10	1	1	1	3	1.000
11	1	1	1	3	1.000
12	1	1	1	3	1.000
13	1	1	1	3	1.000
14	1	1	0	2	0.667
15	1	1	0	2	0.667
16	1	0	1	2	0.667
17	0	1	0	1	0.333
18	1	0	1	2	0.667
19	1	0	0	1	0.333
20	0	1	1	2	0.667
21	1	1	1	3	1.000
22	1	0	0	1	0.333
23	1	0	0	1	0.333
24	1	1	1	3	1.000
25	1	1	1	3	1.000
26	1	0	0	1	0.333
27	0	1	0	1	0.333
28	0	1	1	2	0.667
29	1	0	1	2	0.667
30	0	1	1	2	0.667
31	1	1	0	2	0.667
32	0	1	1	2	0.667
33	1	1	1	3	1.000
34	1	1	1	3	1.000
35	0	1	1	2	0.667
36	0	0	1	1	0.333
37	1	0	0	1	0.333
**Calling probability**					**0.676**
