# Supplementary material for: Density and population size estimates of the endangered northern yellow-cheeked crested gibbon Nomascus annamensis in selectively logged Veun Sai-Siem Pang National Park in Cambodia using acoustic spatial capture-recapture methods
Source: PLoS One. 2023 Nov 27;18(11):e0292386. doi: 10.1371/journal.pone.0292386 (PMC10681233; doi:10.1371/journal.pone.0292386)
Supplement: S4 Table — These data were used to calculate the mean (cm) and standard deviation (cm) values presented in Table 4. (PDF) [file pone.0292386.s004.pdf]

S4 Table. The stump height (cm) and diameter (cm) of the four most logged tree species encountered within 13 sites in Veun Sai-Siem Pang National Park, Cambodia, between January and April 2019. These data were used to calculate the mean (cm) and standard deviation (cm) values presented in Table 4.

	Pterocarpus macrocarpus	*Hopea* spp.	*Sindora cochinchinensis*	*Sterculia lychnophora*	*Pterocarpus macrocarpus*	*Hopea* spp.	*Sindora cochinchinensis*	*Sterculia lychnophora*
**Mean (cm)**	80.32	124.91	88.27	42.23	65.26	126.79	61.38	95.00
**SD (cm)**	44.36	62.50	25.94	14.60	25.16	55.27	25.97	30.27
**N**	56	64	73	66	86	84	74	66
	**Diameter (cm)**				**Stump height (cm)**			
	42.11	124.78	118.86	53.99	67.50	103.00	72.70	100.50
	84.70	124.94	74.17	30.37	39.20	108.00	48.50	92.70
	32.85	117.20	84.83	37.72	64.00	97.60	47.50	85.90
	94.03	102.88	61.59	40.58	79.60	188.20	70.50	71.00
	109.40	105.84	61.97	31.07	60.10	136.00	45.10	102.30
	171.25	69.46	63.66	24.06	91.40	33.50	34.10	93.70
	75.15	68.66	67.29	32.88	62.60	87.40	87.50	53.90
	58.89	73.37	92.82	56.66	84.50	88.70	51.30	95.00
	85.78	74.01	59.43	45.58	63.00	60.30	64.30	87.20
	111.41	129.23	37.75	49.53	82.00	196.10	53.80	80.40
	31.67	127.32	70.76	30.53	60.10	79.80	75.00	89.40
	94.86	219.60	65.89	78.81	16.20	92.50	59.10	101.50
	131.08	118.22	75.15	27.06	99.50	122.30	43.50	92.00
	57.77	117.58	97.28	84.51	86.20	103.30	67.20	79.90
	66.85	111.25	94.28	43.32	76.90	118.30	67.00	91.10
	35.68	79.26	79.51	40.49	14.20	127.40	21.80	89.90
	186.27	74.29	90.15	46.31	117.00	126.50	47.20	173.50
	73.31	71.81	81.52	46.31	65.50	100.80	14.80	103.60
	26.58	89.89	97.12	60.45	23.10	86.20	40.40	102.50
	108.45	77.83	107.08	50.32	64.00	84.50	47.30	85.00
	35.33	74.26	112.78	25.81	132.40	38.10	105.00	125.30
	54.11	83.52	95.72	56.02	65.50	59.20	60.40	92.50
	36.26	78.78	79.96	43.77	55.20	88.20	42.20	78.00
	55.42	66.94	68.91	57.71	49.60	80.10	78.20	200.00
	241.31	79.64	79.70	70.03	98.30	79.30	51.20	209.00
	77.09	78.62	53.60	36.48	58.30	75.40	17.20	87.40
	40.58	119.14	80.56	56.02	40.07	92.20	56.50	111.90
	75.76	124.62	99.47	44.69	121.40	77.20	100.60	95.70
	67.51	110.17	119.08	33.90	57.50	57.10	56.10	100.00
	53.44	79.77	83.14	64.14	61.30	48.50	65.20	85.20
	96.86	67.32	84.16	31.99	57.70	117.90	71.00	98.20
	78.69	113.89	76.78	40.97	71.20	85.10	65.40	72.50
	96.83	83.87	89.19	30.56	32.80	101.50	105.50	84.50
	41.70	122.55	78.97	35.84	9.00	77.60	39.00	72.30
	41.06	228.77	73.21	33.74	61.60	93.30	37.00	66.50
	49.62	103.13	63.66	28.97	54.00	157.70	57.00	73.50
	95.78	102.18	84.48	40.93	51.90	105.90	60.30	91.30
	46.95	209.26	79.90	28.52	62.90	112.00	82.00	93.80
	98.68	142.76	69.39	24.70	67.20	146.00	95.10	85.00
	28.01	103.86	111.89	33.80	74.30	158.20	117.30	101.60
	43.00	141.17	112.68	48.22	57.30	132.20	95.20	95.00

S4 Table. continued.

	Pterocarpus macrocarpus	*Hopea* spp.	*Sindora cochinchinensis*	*Sterculia lychnophora*	*Pterocarpus macrocarpus*	*Hopea* spp.	*Sindora cochinchinensis*	*Sterculia lychnophora*
	**Diameter (cm)**				**Stump height (cm)**			
	92.15	366.31	90.46	37.53	80.00	157.50	100.50	92.50
	72.57	359.85	103.83	42.65	71.00	157.50	59.10	90.00
	95.84	254.87	104.09	43.07	64.40	137.00	82.60	86.50
	175.39	133.09	89.92	40.33	53.20	70.20	70.50	213.10
	104.95	169.50	80.41	57.45	81.20	179.00	75.90	96.40
	90.18	132.35	117.14	81.49	30.50	170.50	107.00	89.30
	143.08	131.84	84.19	27.12	76.50	270.60	67.20	113.00
	81.49	134.49	127.01	26.64	87.20	139.10	26.50	58.40
	47.87	166.51	141.97	28.71	41.20	106.10	47.30	90.00
	57.30	201.08	100.46	33.33	48.20	190.60	88.30	95.60
	38.20	132.42	138.78	49.40	67.10	101.70	20.50	82.50
	59.01	248.38	120.64	42.84	53.00	132.40	37.20	91.20
	53.99	136.55	127.32	31.74	81.10	186.30	39.20	79.50
	82.92	68.02	146.44	28.49	73.50	75.00	115.20	67.40
	171.09	139.55	125.96	28.39	41.50	124.50	60.50	70.10
		178.89	111.69	30.72	75.30	159.60	60.60	65.00
		119.68	46.63	26.58	150.00	224.00	32.70	56.70
		55.90	46.22	31.58	63.40	223.50	85.80	87.10
		80.31	70.19	75.76	38.00	250.70	95.00	116.80
		76.81	69.42	58.73	20.80	229.50	73.50	72.20
		105.46	47.59	36.38	78.00	225.70	58.20	95.20
		109.50	63.09	27.31	44.70	254.50	73.30	99.70
		101.54	70.09	42.08	69.80	206.50	15.20	105.00
			75.92	50.45	82.60	130.90	42.20	68.30
			62.71	30.81	29.50	177.80	12.00	98.30
			85.31		107.20	207.20	96.00	
			72.26		20.10	102.20	31.70	
			113.00		81.40	77.70	36.00	
			99.82		74.90	79.50	15.40	
			152.15		91.30	44.00	90.60	
			137.67		96.20	74.40	85.50	
			43.13		82.10	191.50	52.50	
					94.50	205.50	72.50	
					61.90	123.50		
					64.40	173.60		
					56.70	86.40		
					52.00	166.50		
					63.00	81.50		
					54.50	100.90		
					25.20	68.50		
					70.70	137.30		
					67.00	215.00		
					60.50	111.50		
					57.40			
					73.90			
