# Supplementary material for: Density and population size estimates of the endangered northern yellow-cheeked crested gibbon Nomascus annamensis in selectively logged Veun Sai-Siem Pang National Park in Cambodia using acoustic spatial capture-recapture methods
Source: PLoS One. 2023 Nov 27;18(11):e0292386. doi: 10.1371/journal.pone.0292386 (PMC10681233; doi:10.1371/journal.pone.0292386)
Supplement: S5 Table — These data were used to calculate the mean (cm) and standard error (cm) values presented in Fig 2. (PDF) [file pone.0292386.s005.pdf]

S5 Table. The diameter at breast height (DBH; cm) of all trees (including bamboo) with DBH of ≥ 10 cm within 25 m × 25 m ecological plots (N = 27) in seven sites, located in Veun Sai-Siem Pang National Park, Cambodia. These data were used to calculate the mean (cm) and standard error (cm) values presented in Fig 2.

	Site 1			
Plot	P1	P2	P3	P4
Mean	25.0	24.0	25.6	24.1
SE	2.7	3.0	2.7	4.0
N	24	21	24	35
1	16.8	13.5	14.6	12.4
2	13.2	12.9	32.1	11.7
3	17.8	11.9	17.1	27.0
4	27.1	21.0	37.6	22.7
5	22.5	10.2	40.9	18.8
6	23.7	21.8	38.1	138.8
7	58.3	36.3	17.7	13.9
8	11.3	15.9	16.8	14.1
9	14.8	20.0	31.3	19.0
10	59.7	14.3	61.3	16.3
11	39.7	15.0	15.6	13.7
12	10.7	43.8	11.7	14.0
13	15.8	54.4	22.5	35.0
14	18.3	24.3	12.0	49.0
15	22.8	13.4	37.4	15.8
16	41.8	12.4	24.0	12.1
17	15.5	25.2	16.8	13.8
18	16.8	28.6	49.8	16.5
19	22.6	31.8	29.6	11.8
20	22.9	18.5	26.7	60.9
21	30.0	57.7	19.3	10.9
22	27.5		12.7	18.2
23	36.3		17.5	13.4
24	15.3		10.9	11.8
25				25.3
26				40.8
27				22.9
28				22.0
29				18.7
30				10.0
31				17.1
32				10.7
33				57.3
34				15.4
35				12.7
36				

	Site 2			
Plot	P1	P2	P3	P4
Mean	19.5	23.6	14.6	22.7
SE	3.3	3.8	1.7	5.7
N	19	30	6	13
1	12.6	10.0	15.3	24.1
2	14.6	39.2	22.2	20.5
3	32.9	12.4	14.4	28.5
4	10.8	18.5	14.3	10.3
5	10.8	31.9	10.8	11.1
6	22.7	13.3	10.4	10.0
7	21.8	15.5		13.7
8	23.5	28.0		10.6
9	10.1	18.7		87.5
10	12.6	32.8		11.1
11	10.2	17.5		25.3
12	13.8	21.4		25.9
13	31.8	21.1		16.0
14	71.3	71.2		
15	10.5	23.2		
16	13.0	112.4		
17	18.5	10.2		
18	17.8	13.3		
19	11.8	14.1		
20		14.2		
21		27.5		
22		11.8		
23		27.9		
24		20.7		
25		14.8		
26		11.1		
27		12.0		
28		21.9		
29		11.1		
30		11.3		
31				
32				
33				
34				
35				
36				

	Site 3			
Plot	P1	P2	P3	P4
Mean	36.1	18.3	26.0	30.4
SE	8.4	1.9	2.9	3.9
N	21	21	17	24
1	15.3	19.1	16.5	10.7
2	11.3	36.1	17.8	12.2
3	53.8	16.4	25.8	16.9
4	15.4	11.8	23.3	49.0
5	23.0	10.6	10.2	51.9
6	59.8	10.2	46.3	16.2
7	22.0	12.1	12.0	10.4
8	24.3	20.8	17.6	59.7
9	20.4	13.1	34.6	15.0
10	42.3	12.1	33.5	20.7
11	14.7	23.4	17.0	53.3
12	23.6	11.4	26.0	44.0
13	184.4	14.2	57.6	11.2
14	13.2	28.7	24.4	23.6
15	14.3	12.7	24.2	45.6
16	75.7	21.5	27.4	17.1
17	30.9	42.7	27.5	69.7
18	15.9	22.4		36.7
19	60.5	18.4		15.1
20	18.2	15.4		10.4
21	19.0	12.2		31.2
22				34.1
23				13.1
24				62.7
25				
26				
27				
28				
29				
30				
31				
32				
33				
34				
35				
36				

	Site 5		
Plot	P1	P2	P3
Mean	30.2	17.7	20.0
SE	5.3	2.2	3.0
N	16	16	22
1	20.9	16.3	26.5
2	14.7	20.7	23.4
3	14.7	13.2	17.7
4	79.7	23.4	15.3
5	44.4	41.4	18.6
6	20.4	28.7	76.8
7	23.2	27.7	12.1
8	18.5	14.4	12.8
9	16.7	11.8	17.6
10	17.8	10.9	12.3
11	21.1	10.3	18.3
12	24.3	18.2	13.1
13	16.1	12.1	19.1
14	63.7	11.8	11.2
15	19.3	12.5	11.1
16	68.5	10.3	13.7
17			35.7
18			15.7
19			13.7
20			25.3
21			15.9
22			15.0
23			
24			
25			
26			
27			
28			
29			
30			
31			
32			
33			
34			
35			
36			

	Site 6			
Plot	P1	P2	P3	P4
Mean	21.4	18.8	29.5	22.4
SE	1.5	2.4	5.1	3.7
N	26	19	20	15
1	13.2	12.3	40.7	25.9
2	25.5	14.5	25.1	25.8
3	11.5	41.7	19.2	16.3
4	10.5	13.5	13.5	40.1
5	28.0	14.2	85.8	30.4
6	37.7	13.8	68.4	23.0
7	25.0	28.4	13.8	17.3
8	16.1	47.8	10.0	15.7
9	12.6	12.2	14.6	12.2
10	17.3	17.4	12.1	13.4
11	38.9	11.0	13.5	18.9
12	10.7	19.2	33.4	64.0
13	25.6	16.1	15.4	10.5
14	25.2	18.6	17.3	11.9
15	12.4	25.0	17.3	10.2
16	27.2	12.0	29.9	
17	21.0	15.4	15.1	
18	20.4	11.7	17.2	
19	17.8	12.3	61.8	
20	30.6		65.7	
21	23.2			
22	17.5			
23	16.3			
24	23.4			
25	19.9			
26	28.4			
27				
28				
29				
30				
31				
32				
33				
34				
35				
36				

	Site 7			
Plot	P1	P2	P3	P4
Mean	23.4	37.7	11.1	11.9
SE	2.8	14.8	0.5	0.4
N	30	13	30	36
1	14.0	10.3	11.0	10.6
2	23.4	10.2	10.2	11.7
3	12.9	12.1	10.3	10.6
4	13.1	10.2	11.1	11.4
5	80.4	10.8	10.2	10.1
6	53.0	58.4	12.0	10.8
7	19.9	179.0	10.0	11.1
8	37.4	17.5	10.2	10.0
9	11.6	124.2	10.2	10.5
10	34.1	24.7	10.3	15.0
11	16.6	10.9	10.6	12.1
12	22.0	11.1	10.8	11.5
13	22.3	10.2	10.6	10.3
14	26.4		10.0	10.0
15	18.6		10.5	10.7
16	12.5		10.3	10.3
17	10.2		10.0	19.3
18	15.6		10.7	15.0
19	10.6		10.9	20.1
20	44.8		10.0	15.1
21	12.5		11.3	10.4
22	18.5		22.5	10.5
23	11.3		10.5	10.5
24	38.8		10.0	10.5
25	17.6		10.7	10.2
26	23.7		10.2	10.5
27	27.1		10.2	10.2
28	26.1		16.7	14.6
29	17.4		10.4	10.0
30	10.0		10.2	14.6
31				10.2
32				10.5
33				11.8
34				14.0
35				11.1
36				11.0

	Site 12			
Plot	P1	P2	P3	P4
Mean	27.6	15.9	32.4	24.7
SE	4.8	1.1	6.4	4.8
N	16	33	17	20
1	12.0	20.1	34.5	10.8
2	21.6	19.9	61.8	40.6
3	12.6	10.0	25.8	20.4
4	11.6	10.4	30.4	24.4
5	23.4	19.3	70.7	20.2
6	73.2	12.8	43.8	28.6
7	28.2	24.3	12.1	13.6
8	15.2	14.6	13.2	17.1
9	19.1	11.6	107.0	48.1
10	24.1	11.0	14.3	10.5
11	19.0	10.2	37.9	33.9
12	44.4	10.1	36.9	10.2
13	20.9	10.3	19.8	11.0
14	30.5	10.5	12.3	101.2
15	16.1	28.8	10.5	20.1
16	70.0	21.5	10.2	38.2
17		24.2	10.1	12.2
18		12.4		10.0
19		10.3		11.6
20		10.0		10.9
21		10.1		
22		12.1		
23		21.4		
24		12.8		
25		10.9		
26		33.4		
27		23.5		
28		14.5		
29		14.2		
30		14.8		
31		22.1		
32		22.4		
33		11.2		
34				
35				
36				
