# Supplementary material for: Density and population size estimates of the endangered northern yellow-cheeked crested gibbon Nomascus annamensis in selectively logged Veun Sai-Siem Pang National Park in Cambodia using acoustic spatial capture-recapture methods
Source: PLoS One. 2023 Nov 27;18(11):e0292386. doi: 10.1371/journal.pone.0292386 (PMC10681233; doi:10.1371/journal.pone.0292386)
Supplement: S6 Table — These data were used to determine the frequency of the first duets (N = 168) from gibbon groups heard during 10-minute blocks relative to sunrise in the auditory survey period in Veun Sai-Siem Pang National Park, Cambodia, presented in Fig 3. The frequency of first duets (x) in category ‘0’ are those that began -10 < x ≤ 0 minutes relative to sunrise. (PDF) [file pone.0292386.s006.pdf]

S6 Table. The start time of the first duet call heard from each *N. annamensis* group relative to sunrise. These data were used to determine the frequency of the first duets (N = 168) from gibbon groups heard during 10-minute blocks relative to sunrise in the auditory survey period in Veun Sai-Siem Pang National Park, Cambodia, presented in Fig 3. The frequency of first duets (x) in category '0' are those that began $-10 < x \leq 0$ minutes relative to sunrise.

Category	-40	-30	-20	-10	0	10	20	30	40	50	60	70	80	90	100	110	120
N	*3*	*5*	*17*	*32*	*40*	*26*	*12*	*10*	*5*	*4*	*0*	*0*	*4*	*2*	*4*	*3*	*1*
	**Start time of first duet call relative to sunrise (minutes)**																
**1**	-43.73	-36.67	-29.40	-19.20	-9.97	1.28	10.08	20.92	30.07	44.88			70.67	80.63	91.53	101.17	110.27
**2**	-43.00	-35.98	-27.70	-18.33	-9.97	2.00	10.65	21.83	32.00	46.70			74.83	83.83	95.37	103.53	
**3**	-40.83	-31.55	-27.35	-17.90	-9.50	2.33	10.68	23.00	33.15	49.80			75.07		97.00	104.47	
**4**		-31.00	-27.17	-16.95	-9.17	2.83	12.45	23.65	34.00	50.00			79.17		98.67		
**5**		-30.33	-26.10	-16.10	-8.47	3.05	13.40	24.25	37.53								
**6**			-25.88	-16.05	-8.33	3.22	13.43	24.60									
**7**			-25.42	-15.55	-8.00	3.67	13.97	25.85									
**8**			-25.08	-15.50	-7.53	3.67	14.35	26.32									
**9**			-24.82	-15.17	-7.42	4.00	16.33	29.00									
**10**			-24.08	-15.00	-7.20	4.00	16.83	29.05									
**11**			-23.87	-14.17	-7.00	4.17	18.00										
**12**			-23.00	-14.08	-6.78	4.27	20.00										
**13**			-22.55	-14.00	-6.78	4.87											
**14**			-22.35	-13.90	-6.68	4.93											
**15**			-21.50	-13.82	-6.65	6.00											
**16**			-21.18	-13.67	-6.47	6.60											
**17**			-20.00	-13.33	-5.70	7.00											
**18**				-12.68	-4.47	7.33											
**19**				-12.33	-4.45	7.50											
**20**				-11.80	-4.43	7.97											
**21**				-11.67	-4.00	8.12											
**22**				-11.50	-3.67	8.18											
**23**				-11.27	-3.33	8.60											
**24**				-11.17	-3.33	8.67											
**25**				-11.00	-3.17	9.00											
**26**				-10.87	-3.00	9.00											
**27**				-10.83	-3.00												
**28**				-10.83	-3.00												
**29**				-10.72	-3.00												
**30**				-10.53	-2.90												
**31**				-10.50	-2.77												
**32**				-10.43	-2.65												
**33**					-2.00												
**34**					-1.77												
**35**					-1.00												
**36**					-0.72												
**37**					-0.67												
**38**					-0.50												
**39**					0.00												
**40**					0.00												
